# Cost-utility analysis on robot-assisted and laparoscopic prostatectomy based on long-term functional outcomes

**DOI:** 10.1038/s41598-022-10746-3

**Published:** 2022-05-10

**Authors:** Melanie A. Lindenberg, Valesca P. Retèl, Henk G. van der Poel, Ferdau Bandstra, Carl Wijburg, Wim H. van Harten

**Affiliations:** 1 Division of Psychosocial Research and Epidemiology, Antoni van Leeuwenhoek, Amsterdam, The Netherlands; 2 Department of Urology, Antoni van Leeuwenhoek, Amsterdam, The Netherlands; 3 Department of Health Technology and Services Research, University of Twente, MB-HTSR, PO Box 217, 7500 Enschede, The Netherlands; 4 Vrije Universiteit (VU), Amsterdam, The Netherlands; 5 Department of Urology Rijnstate Hospital, Arnhem, The Netherlands

**Keywords:** Surgical oncology, Prostate, Health care economics

## Abstract

Robot-Assisted Radical Prostatectomy (RARP) is one of the standard treatment options for prostate cancer. However, controversy still exists on its added value. Based on a recent large-sample retrospective cluster study from the Netherlands showing significantly improved long-term urinary functioning after RARP compared to Laparoscopic RP (LRP), we evaluated the cost-effectiveness of RARP compared to LRP. A decision tree was constructed to measure the costs and effects from a Dutch societal perspective over a ~ 7 year time-horizon. The input was based on the aforementioned study, including patient-reported consumption of addition care and consumed care for ergonomic issues reported by surgeons. Intervention costs were calculated using a bottom-up costing analysis in 5 hospitals. Finally, a probabilistic-, one-way sensitivity- and scenario analyses were performed to show possible decision uncertainty. The intervention costs were €9964 for RARP and €7253 for LRP. Total trajectory costs were €12,078 for RARP and €10,049 for LRP. RARP showed higher QALYs compared to LRP (6.17 vs 6.11). The incremental cost-utility ratio (ICUR) was €34,206 per QALY gained, in favour of RARP. As a best-case scenario, when RARP is being centralized (> 150 cases/year), total trajectory costs decreased to €10,377 having a higher utilization, and a shorter procedure time and length of stay resulting in an ICUR of €3495 per QALY gained. RARP showed to be cost-effective compared to LRP based on data from a population-based, large scale study with 7 years of follow-up. This is a clear incentive to fully reimburse RARP, especially when hospitals provide RARP centralized.

## Introduction

Radical prostatectomy is recommended as one of the front-line treatments for men diagnosed with localized prostate cancer who have a life expectancy greater than 10 years^1,2^. In many countries, this procedure is currently performed using Robot-Assisted Radical Prostatectomy (RARP), showing improvements compared to Open (ORP) and Laparoscopic (LRP) radical prostatectomy in urinary incontinence, erectile functioning, hospital stay, and blood loss^3–5^, but showing no benefits on oncological outcomes^6^. Additionally, RARP showed improved ergonomics compared to ORP and LRP^7^. However, based on the current evidence base, systematic reviews and meta-analyses concluded that the quality of the evidence is too limited to draw definite conclusions on the advantages of RARP compared to LRP^8–11^. For the Dutch National Health Care Institute and many other national reimbursement bodies, this is the reason to reimburse RARP not for its actual costs but for costs of ORP or LRP. Therefore, hospitals are faced with substantial additional costs, money that otherwise could be used for improvements in quality of care within a hospital.

Aiming at filling this research gap, a retrospective cluster study was conducted evaluating real-world data from 12 hospitals in the Netherlands (n = 1370) to evaluate long-term (median follow-up of 7.08 years) functional and oncologic outcomes and besides evaluate perioperative outcomes, and healthcare usage^12^. This study showed similar survival and oncologic outcomes, but better perioperative outcomes and significantly improved urinary functioning after RARP compared to LRP.

As a part of this retrospective cluster study, the present analysis aimed to comprehensively evaluate the intervention costs of RARP and LRP, and evaluate the cost-effectiveness of RARP compared to LRP from a Dutch societal perspective.

## Methods

### Research design and study sample

The design of this study follows the aforementioned retrospective cluster study^12^. In total 1370 patients were included undergoing either RARP or LRP between 2010 and 2012 in 12 hospitals in the Netherlands^12^. In this study, data were collected at one moment in time at least 5 years after surgery.

A decision tree was constructed in Microsoft Excel (Supplement A) starting with prostate cancer patients undergoing RARP or LRP. As no significant differences in oncologic outcomes and prostate cancer-specific survival were found^12^, the analysis focussed on functional outcomes. After RARP and LRP, patients could end up in the following health states: “continent and potent”, “continent and impotent” and “incontinent and impotent”.

The analysis was performed from a societal perspective in the Netherlands and the time horizon corresponds with the median follow-up period of 7.08 (range: 5.27 – 9.86) years^12^.

### Input parameters

All input parameters are presented in Table 1.

**Table 1:** Input parameters for the cost-effectiveness analysis.

Input parameters	RARP		LRP		Distribution	Source
Parameter name	Det value	SE	Det Value	SE		
Probability						
Of being in a certain health state						
“continent and potent”	13.03%	0.012	5.25%	0.011	Dirichlet	^12^
“continent and impotent”	55.64%	0.017	45.05%	0.025	Dirichlet	^12^
“incontinent and impotent”	31.33%	0.016	49.70%	0.025	Dirichlet	^12^
Of having complications after surgery						
Clavien-Dindo grade 1	7.71%	0.010	7.33%	0.014	Beta	^12^
Clavien-Dindo grade 2	4.51%	0.007	2.80%	0.009	Beta	^12^
Clavien-Dindo grade 3	5.07%	0.008	4.53%	0.011	Beta	^12^
Clavien-Dindo grade 4	1.20%	0.004	1.72%	0.007	Beta	^12^
Of receiving home care after surgery						
Receiving home care	1.80%	0.004	3.50%	0.009	Beta	^12^
Hours per week (mean)	7.03	2.54	5.5	2.35	Gamma	^12^
Number of weeks (mean)	4.2	0.85	8.5	3.5	Gamma	^12^
Of receiving additional care for incontinence complaints after surgery						
Receiving physiotherapy	42.5%*	0.023	58.5%*	0.023	Beta	^12^
Number of visits (mean)	7.85	0.315	9.40	0.519	Gamma	^12^
Consulting a General Practitioner (GP)	2.7%*	0.008	3.7%*	0.009	Beta	^12^
Number of visits (mean)	3.40	0.852	5.88	2.377	Gamma	^12^
Sphincter placement	2.5%*	0.007	8.6%*	0.013	Beta	^12^
Of number of pads used in the “incontinent and impotent” health state (measured at follow-up)						
1 pad	61.2%	0.017	61.3%	0.025	Beta	^12^
2 pads	23.3%	0.015	20.9%	0.021	Beta	^12^
3 or more pads	15.5%	0.013	17.8%	0.019	Beta	^12^
Of receiving additional care for complaints of erectile dysfunction after surgery						
Receiving physiotherapy	2.31%	0.005	2.91%	0.0078	Beta	^12^
Number of visits (mean)	6.72	1.03	8.67	1.79	Gamma	^12^
Consulting a General Practitioner (GP)	3.59%	0.006	2.41%	0.007	Beta	^12^
Number of visits (mean)	2.88	0.44	2.00	0.23	Gamma	^12^
Consulting a different specialist	10.11%	0.010	13.4%	0.016	Beta	^12^
Number of visits (mean)	3.80	0.78	3.29	0.38	Gamma	^12^
Place a prosthesis	0.32%	0.004	0%	0.000	Beta	^12^
Use a vacuum constriction device	4.11%	0.012	5.26%	0.019	Beta	^12^
Of receiving pharmaceuticals for erectile dysfunction after surgery and during follow-up						
Initial use of an PDE-5 inhibitor	16.73%	0.016	15.46%	0.023	Beta	^12^
Success rate PDE-5 inhibitor	37%	0.037	27%	0.035	Beta	^12^
Continuous use of an PDE-5 inhibitor†	6.1%	–	4.2%	–	–	–
Initial use of ICI	9.94%	0.004	9.67%	0.022	Beta	^12^
Success rate ICI	70%	0.036	70%	0.036	Beta	estimation [A]
Continuous use of ICI†	6.95%	–	6.77%	–	–	–
Initial use of IUI	0.47%	0.016	2.46%	0.014	Beta	^12^
Success rate IUI	56%	0.029	56%	0.029	Beta	[B]
Continuous use of an IUI †	0.27%	–	1.38%	–	–	–
Frequency per year (PDE-5 inhibitor)	156	–	156	–	–	EAU Guidelines
Frequency per year (IUI and ICI)	104	–	104	–	–	EAU Guidelines
Frequency for initial use	5		5			Expert opinion

Utility values	RARP and LRP	SE				
“continent and potent”	0.9638 (n = 149)	0.01			Beta	^12^
“continent and impotent”	0.9309 (n = 904)	0.00			Beta	^12^
“incontinent and impotent”	0.8964 (n = 169)	0.01			Beta	^12^

Cost parameters	RARP		LRP			
	Det value	SE	Det value	SE		
Intervention costs†	€9,963.71	€147.24	€7,253.36	€182.62	Gamma	See Table 2
State costs incontinence in ”incontinent and impotent” (pad use)#	€2,086.2	€266.09	€2,115.9	€269.89	Gamma	–
State costs for having complaints of erectile dysfunction (medicine use)#	€1,076.9	€137.36	€1021.5	€130.29	Gamma	–

Cost parameters (unit costs)	RARP and LRP	SE	Distribution	Source		
Receiving homecare (per hour)	€ 65.68	€ 8.378	Gamma	^13^		
Costs complication grade 1	€ 579.39	€ 73.902	Gamma	Expert opinion – ^13,14^ ‡		
Costs complication grade 2	€ 1,158.79	€ 147.801	Gamma	Expert opinion – ^13,14^ ‡		
Costs complication grade 3	€ 3,949.85	€ 503.81	Gamma			
Costs complication grade 4	€ 10,760.18	€ 1372.47	Gamma			
Costs of one pad	€ 0.52	€ 0.07	Gamma	Abena Man (Dutch company)		
Physiotherapy consult	€ 35.24	€ 4.495	Gamma	^13^		
GP consult	€ 35.24	€ 4.495	Gamma	^13^		
Consult with a specialist	€ 117.59	€ 14.99	Gamma	^13^		
Sphincer placement	€ 2,455.00	€ 313.14	Gamma	∆		
Prothesis placement	€ 10,003.17	€ 1275.91	Gamma	Π		
Vacuum constrictor	€ 250.00	€ 31.89	Gamma	[C]		
PDE-5 inhibitor (50 mg)	€ 8.06	€1.03	Gamma	[D]		
ICI (Phentolamine /​papaverine 15 mg/0.5 ml)	€ 9.80	€1.250	Gamma	[D]		
IUI first time (alprostadil, 1000 μg)	€ 15.36	€1.959	Gamma	[D]		

Parameters for including use of care by surgeons and sick-leave of surgeons due to pain complaints						
	RARP		LRP		Distribution	Source
	Det value	SE	Det Value	SE		
Additional care used by surgeons because of neck and/or back complaints						
% used additional care for pain complaints	7.14%	0.066	21.40%	0.106	Beta	Supplement B and C
Frequency of care used	5	0.640	5	0.640	Gamma	Supplement B and C
Total costs for additional care per treatment arm	€ 294.42		€ 475.16			Section 2.3
Sick leave of surgeons because of neck and/or back complaints (friction cost method)						
Surgeons with sick leave because of pain complaints	1.00%	0.026	7.14%	0.066	Beta	Supplement C
Proportion surgical activities of total work activities (% to replace when sick)	20%		20%		–	Supplement C
Duration of sick leave (weeks)	10	1.28	10	1.28	Gamma	Supplement C
Frequency of sick leave	2	0.26	2	0.26	Gamma	Supplement C
Friction period	12.6	–	12.6	–	–	^13^
Costs per hour	€124	–	€124	–	–	^13^
Friction costs	€99,111.2	–	€99,111.2	–	–	Replaced for 20%
Total costs per treatment arm	€991.1		€7,076.5			Section 2.3
Additional modelling parameters						
Discounting rate costs	0.04					^13^
Discounting rate QALYs	0.015					^13^
Time horizon	7.08 years					^12^

Det. Value = Deterministic value, ICI = Intra-cavernous injection, IUI = intra-urethral injection, GP = General practitioner.

[A] Coombs et al. 2012 A review of outcomes of an intracavernosal injection therapy programme; [B] Guay et al. 2000 Clinical experience with intraurethral alprostadil (MUSE) in the treatment of men with erectile dysfunction. [C] University hospital of Ghent – patient brochure; [D] Zorginstituut Nederland Pharmacy costs available from www.medicijnkosten.nl.

*Shows the percentages of patients that used an additional type of care of the whole population. For this purpose, the percentages based on Lindenberg et al. 2021 (Table 1) describing the use of additional care and having complaints were multiplied. † More information on calculation of this parameter is presented in Table 2. # These cost are the result of combining the percentages of pads use per intervention and the unit costs, and combining the percentages of continuous use (initial use multiplied with the success rate) of an PDE-5 inhibitor, ICI, IUI with the unit costs of the pharmaceuticals. † Continuous use was found by multiplying the initial use times the success rates.

‡DRG code 182,199,024 ∆ DRG code 149,999,079 Π DRG code 149,899,005 The costs for the DRGs were retreived from https://www.opendisdata.nl/msz/zorgproduct (Dutch website).

### Transition probabilities

To define whether a patient ended-up in a certain health state the following definitions were used: patients using no pads (EPIC-26 question 27) were considered continent, patients having a score of ≥ 17 on the Sexual Health Inventory for Men (SHIM) questionnaire were considered potent. Since no cut-off value is known for the EPIC-26 Sexual domain (primary outcome of the retrospective cluster study) to define patients having erectile dysfunction, the SHIM questionnaire was also included in the survey^12^. Supplement B shows the observed scores on the SHIM. The analysis assumed that patients were in those states for the complete time horizon.

As the combination of being incontinent and potent was not common according to our experts and this group was too small to perform separate analyses on (2.6%), this combination was not taken into account.

We also incorporated the risk of having complications, receiving homecare after surgery, use of additional care for incontinence and erectile dysfunction complaints directly after surgery (e.g. physiotherapy, sphincter placement), and for a longer period (e.g. pad use and pharmaceuticals)^12^.

### Utility values

Utilities, values between 0 and 1 where a higher score indicates better health, were evaluated by the EQ5D-5L questionnaire. For each health state, a utility value was calculated (Table 1). The utility value was assumed to be stable over the follow-up period. The utility values were multiplied with the median follow-up time of 7.08 years to obtain the Quality Adjusted Life Years (QALYs).

### Surgeon effects

As part of the retrospective study, a questionnaire (Supplement C) was distributed among surgeons (n = 20) that operated in the selected hospitals between 2010 and 2012 evaluating complaints of back and neck pain after or related to LRP and RARP. Supplement D shows the results of the questionnaire, and Supplement E describes how these effects were translated in monetary values to incorporate the effects in the analysis per treatment arm.

### Intervention costs

The intervention costs were evaluated bottom-up by an Activity-Based Costing (ABC) analysis in 5 hospitals, 2 performing LRP, and 3 performing RARP^15^. The following cost categories were included: personnel, material, use of the OR, medical devices, hospitalization, and overhead costs. Because an additional lymph node dissection (LND) resulted in a longer procedure time, and the percentage differed between interventions^12^, the costs were calculated with and without LND. The cost categories personnel, material, and medical devices were evaluated per hospital. The costs for using the OR were based on a previous study from a Dutch perspective^16^. The hospitalization costs were calculated by taking the average length of stay per intervention multiplied with the reference costs for an admission day^13^. Finally, a weighted mean of the intervention costs with and without LND was calculated^12^. Table 2 shows the input parameters for the intervention costs. In Supplement E more detailed information for the calculation of several cost categories (e.g. health state costs, homecare costs) is provided.

**Table 2:** Intervention costs.

Intervention costs input	RARP (95% CI)	LRP (95% CI)	Source
**Input for RP without LND**			
Procedure time (mean hours)	3.47 (3.37–3.56)	3.61 (3.53–3.69)	^12^
Skin-to-skin procedure time (mean hours)	2.77 (2.68–2.85)	3.06 (2.99–3.12)	^12^
Length of stay (mean days)	3.25 (3.13–3.38)	2.99 (2.86–3.13)	^12^
**Input for RP with LND**			
Procedure time (mean hours)	3.67 (3.54–3.80)	4.25 (4.07–4.42)	^12^
Skin-to-skin procedure time (mean hours)	2.98 (2.87–3.10)	3.74 (3.60–3.88)	^12^
Length of stay (mean days)	3.24 (3.02–3.45)	4.59 (4.03–5.14)	^12^
**Input regardless of with or without LND**			
% receiving LND	37.9% (35%-41%)	26.8% (23%-31%)	^12^
Costs of OR usage per hour	€ 238.20	€ 238.20	^16^
Personnel costs per hour: Anaesthetist (0.5), Surgeon (1–2), OR assistant (2.2), Medical assistant (1) on average per hour	€ 323.66	€ 366.60	Real time observation and [A]
Hospitalization costs per day	€ 505.32	€ 505.32	^13^

Intervention costs results	RARP	LRP	Source/calculation
**Intervention cost without LND (1 procedure)**			
Personnel costs	€ 1,036.17 (10%)	€ 1,225.25 (18%)	^17^
Costs for OR usage	€ 825.90 (8%)	€ 859.88 (12%)	^16^
Hospitalization costs	€ 1,643.87 (17%)	€ 1,512.97 (22%)	^13^
Material costs (e.g. surgical tools, suture material, Da Vinci materials)	€ 2,786.85 (28%)	€ 2,417.67 (35%)	LRP: ^8^*; RARP: based on internal costs per hospital
Medical devices costs (equipment costs and service costs)	€ 2,571.22 (26%)	-	Interviews/internal cost information of 3 hospitals
Overhead costs	€ 1,059.00 (11%)	€ 918.71 (13%)	^8,13^
**Intervention cost with LND (1 procedure)**			
Personnel	€ 1,103.53 (11%)	€ 1,459.54 (18%)	[A]
Costs for OR usage	€ 874.53 (9%)	€ 1,011.32 (12%)	^16^
Hospitalization costs	€ 1,635.30 (16%)	€ 2,317.08 (29%)	^13^
Material costs (e.g. surgical tools, suture material, Da Vinci materials)	€ 2,786.85 (28%)	€ 2,417.67 (30%)	For LRP: ^8^*; RARP based on internal costs
Medical devices costs (equipment costs and service costs)	€ 2,571.22 (26%)	-	Internal cost information
Overhead costs	€ 1,059.00 (11%)	€ 918.71 (11%)	^8,13^
**Total costs without LND**	€ 9,923.01	€ 6,934.48	
**Total costs with LND**	€ 10,030.42	€ 8,124.32	
**Total costs per intervention (used in the CUA)**	€ 9,963.71	€ 7,253.36	

*Exchange rate from pound to euro of 1.23 EUR (average rate of 2012) costs were corrected for inflation (1.105 from 2012 to 2019) [A] Dutch Federation of Academic Medical Centers. Collective labor agreement 2018–2020 for academic medical centers. Utrecht; 2018Dutch Federation of Academic Medical Centers.

### Costs of additional care directly after surgery

Costs for complications were based on expert opinion and a previous evaluation by National Institute for Health and Care Excellence^14^. For homecare costs, a weighted average of the unit costs for personal care, and nursing care was calculated^13^.

For costs using additional care for complaints of incontinence and erectile dysfunction after surgery, the activities and/or pharmaceuticals taking into account the duration and/or frequency of activities were linked to unit costs or costs for DRGs which were corrected for inflation^13,18^ (Table 1). For pharmaceuticals, an initial starting dose of 5 tablets or injections was assumed based on expert opinion.

### Health state costs

The health state costs included the use of pads and pharmaceuticals used for erectile dysfunction complaints (see Supplement E for more information).

### Analysis and sensitivity analyses

In the analysis, the costs were discounted at a rate of 4%, and effects at a rate of 1.5% according to Dutch guidelines. The outcome of the decision tree is the incremental cost-utility ratio (ICUR) calculated by dividing the incremental costs by the incremental QALYs. Furthermore, a Deterministic Sensitivity Analysis (DSA) and a Probabilistic Sensitivity Analysis (ProbSA) were performed to evaluate the impact of parameter uncertainty. For the DSA, all parameters were varied over their upper and lower limits to evaluate the impact on the ICUR. Besides, two different definitions of having no erectile dysfunction (SHIM > 22) and being continent (0–1 pad used) were evaluated.

For the ProbSA, Table 1 shows the distributions used for the parameters in the Monte Carlo simulation (drawing 1000 random samples). All potential outcomes are plotted in a cost-effectiveness (CE-) plane. Furthermore, cost-effectiveness acceptability curves (CEAC) were drafted, indicating the probability that RARP is cost-effective compared to LRP given a certain Willingness To Pay (WTP) ratio. In the Netherlands, the informal WTP ratio is €80,000 per QALY^19^.

### Scenario analysis

Finally, in a scenario analysis, three scenarios were evaluated. The first scenario evaluated the best-case scenario (centralization) by evaluating data from the two hospitals performing > 150 RARPs per year, including potential effects on clinical outcomes. Supplement F shows the detailed calculation and input used for this scenario. In the second scenario, the same intervention costs were included but the potential improved clinical outcomes were not taken into account as the accompanied study showed no linear relationship between hospital volume and improved functional outcomes^12^. In the third scenario, the Da Vinci robot was also used for other indications, evaluating the ICUR over a range of 100 to 850 procedures a year, by only adjusting the medical device costs.

### Ethics approval and consent to participate

The study was approved by the medical ethical committee of the Netherlands Cancer Institute and was judged as a “non-WMO-applicable” research. Patients completed an informed consent form, which explained how their data would be used and reported. The study was performed in accordance with the Declaration of Helsinki.

### Consent for publication

Not applicable.

### Reporting guidelines

The CHEERS guideline was used.

## Results

### Base case analysis results

Total intervention costs were €9,964 for RARP and €7,253 for LRP. The categories medical devices (26%) and material (28%) contributed the most to the intervention costs of RARP. For LRP, the categories material (30%-35%), personnel (18%), and hospitalization (22%-29%) contributed the most.

Total trajectory costs were €12,078 for RARP and €10,049 for LRP. Regarding the follow-up costs, incontinence complaints accounted for the largest difference between LRP and RARP (€629) (Table 3). Total QALYs found for RARP were 6.17 and 6.11 after LRP. Showing incremental costs of €2,029 and incremental QALYs of 0.059 for RARP. RARP shows to be cost-effective at an ICUR of €34,206 as this is below the informal WTP threshold of €80,000 (Table 4).

**Table 3:** Costs per category resulting from the base case analysis (per patient) (not discounted).

	RARP	LRP	Difference
Surgery	€ 9,963.71	€ 7,253.36	€ 2,710.36
Complications after surgery	€ 426.61	€ 439.05	− € 12.43
Home care after surgery	€ 34.91	€ 107.22	− € 72.31
Additional care and sick leave of surgeons	€ 2.33	€ 18.57	− € 16.25
Incontinence complaints after surgery	€ 181.54	€ 412.73	− € 231.19
Complaints regarding erectile dysfunction after surgery	€ 108.16	€ 89.59	€ 18.57
Costs for being incontinent over the total time horizon	€ 653.68	€ 1,051.55	− € 397.87
Costs for having complaints related to erectile dysfunction over the total time horizon	€ 936.57	€ 967.83	− € 31.26
Not discounted total costs	€ 12,307.52	€ 10,339.90	€ 1,967.62
Discounted total costs	€ 12,078.01	€ 10,048.73	€ 2,029.28

**Table 4:** The deterministic results are presented for both the base case analysis and the scenario analysis evaluating a centralization scenario with and without potential clinical improvements because of centralization of care.

	Costs	QALYs	iCosts	iQALY	ICUR
**Deterministic results from the base case analysis**					
RARP	€ 12,078.01	6.17			
LRP	€ 10,048.73	6.11			
			€ 2,029.28	0.059	€ 34,206.26
**Deterministic results from the centralization scenario (scenario 1)**					
RARP	€ 10,377.21	6.20			
LRP	€ 10,048.73	6.12			
			€ 328.48	0.094	€ 3,495.36
**Deterministic results from the centralization scenario without taking into account potential clinical benefits (scenario 2)**					
RARP	€ 10,599.91	6.17			
LRP	€ 10,048.73	6.11			
			€ 551.18	0.059	€ 9,290.88

### Sensitivity analyses

**Figure 1:**
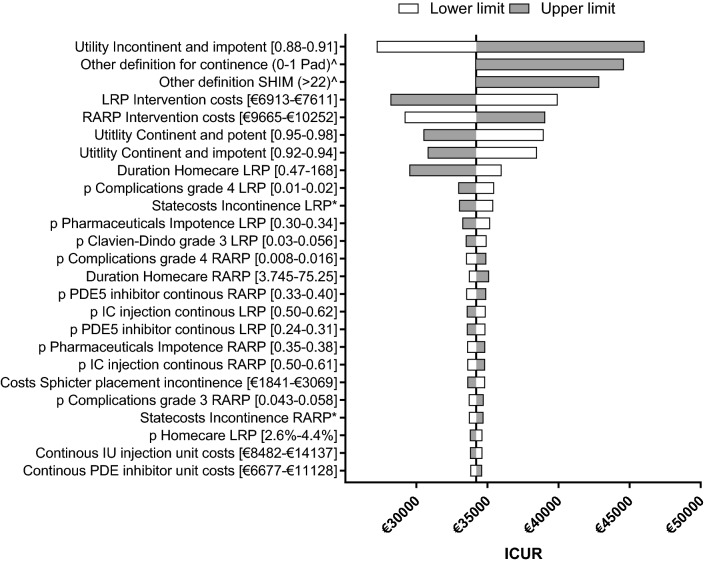
Results from the one-way sensitivity analysis. This figure presents the results of the deterministic one-way sensitivity analysis. This figure shows the influence of the observed uncertainty (lower and upper value) surrounding a specific parameter on the main outcome measure. All parameters starting with a “*p*” indicate a probability. From this figure we learn that the uncertainty surrounding the intervention costs, definitions and utility value showed the largest deviation from the base case ICUR. However this uncertainty does not affect our conclusion. ICUR = incremental cost-utility ratio. * the uncertainty from this parameter was a combined value, the uncertainty surrounding the chance of using 1, 2 and 3 or more pads were changed at the same time. The SE surrounding these parameters can be found in Table 1.

Figure 1 shows that the ICUR was most sensitive to uncertainty surrounding the utility values, intervention costs, and the two other definitions used. Although using another definition for incontinence (€44,596) and erectile dysfunction (€42,867) would show a substantial higher ICUR, it did not alter our conclusion. Uncertainty surrounding other parameters such as surgeon effects and additional care used for incontinence and erectile dysfunction had a limited effect.

**Figure 2:**
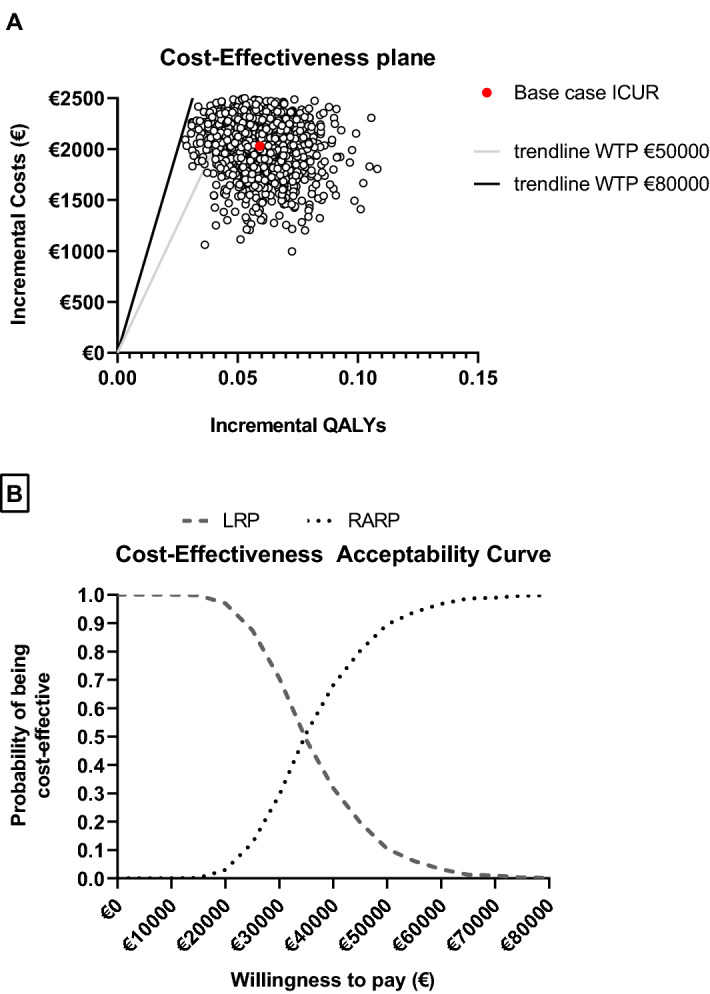
Results from the probabilistic sensitivity analysis. (**a**) presents all potential outcomes given the distribution surrounding the parameter. The trend lines show the WTP thresholds. All potential outcomes are below the WTP threshold of €80,000. The majority of outcomes also fall below the WTP threshold of €50,000. (**b**) shows the probability of RARP being cost-effective, given a certain WTP threshold. The probability of RARP being cost-effective at a WTP threshold of €80,000 is 99.8%.

The ProbSA showed that all possible outcomes indicate that RARP is more effective at higher costs (Fig. 2). According to the CEAC, RARP had a 99.8% probability to become cost-effective at a WTP threshold of €80,000.

### Scenario analyses

**Figure 3:**
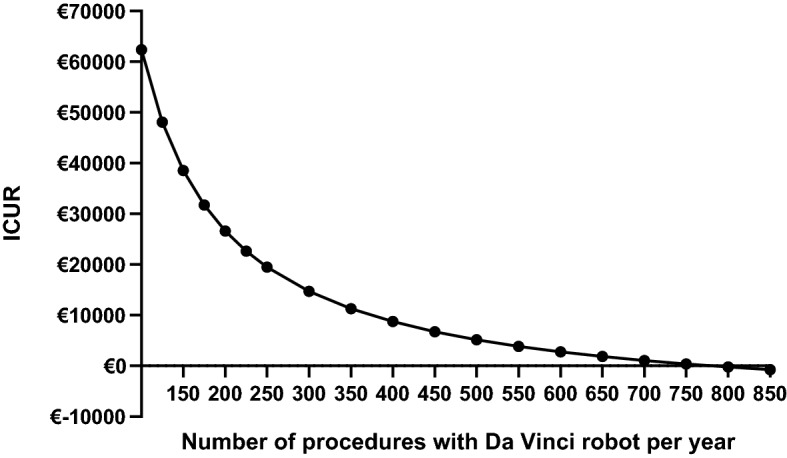
Results from scenario 3. This figure presents the incremental cost-utility ratio (ICUR) when the Da Vinci is used more often. For example when also used for other indications. Showing an ICUR below €20,000 when ≥ 250 procedures are performed per year with the Da Vinci robot. When the robot is fully used, RARP even shows the potential to be cost-saving compared to LRP.

Table 4 shows the results of scenario 1 and 2. Total trajectory costs of scenario 1 were €10,377 and we found 6.20 QALYs for RARP, resulting in an ICUR of €3,495. For scenario 2, we found total trajectory costs of €10,600 and 6.17 QALYs, resulting in an ICUR of €9,291. Figure 3 shows that when a hospital performs ≥ 250 procedures with the Da Vinci robot, the ICUR comes below €20,000, when a hospital has ≥ 800 procedures a year, RARP is becoming cost-saving compared to LRP.

## Discussion

RARP showed to be cost-effective compared to LRP when evaluating long-term functional outcomes, presenting an ICUR of €34,206. These results strengthen the conclusions from the clinical study showing that RARP was more effective compared to LRP on the long-term^12^. These results can be used to inform reimbursement decisions of RARP.

The costs found for RARP (€9,964) and LRP (€7,253) were in line with previously published estimates^20,21^. Compared to LRP, the OR costs, personnel costs, and hospitalization costs were lower for RARP due to shorter procedure times and length of stay. In evaluating the intervention costs of RARP we created a rather negative scenario by assuming the use of the Da Vinci robot only for prostatectomies, although many hospitals use the robot in multiple indications where it also suggests to be cost-effective^22,23^. When increasing the utilization of the robot, the ICUR decreased substantially because of lower per-patient costs as seen in the scenario analysis. Based on our data, centralization of RARP (Table 4) resulted in a decreased length of stay, shorter procedure times, and better outcomes, as has been suggested by literature^24^. We should mention that these scenarios represent a best case example: results from a large volume hospital (> 150 procedures/year) and experienced surgeons, showing ICURs between €3,495 and €9,291. The effect of centralization on the cost-effectiveness may even be underestimated because we evaluated data from the early introduction phase of the Da Vinci robot^25^ and outcomes are expected to improve with surgeon experience^26,27^. Finally, as the material costs are a large driver of the intervention costs, critical appraisal of the instruments used per surgery may be useful. This could result in a cost reduction of ~ €250 per surgery^28^, with substantial influence on the cost-utility (Fig. 1).

The influence of surgeon effects on the cost-effectiveness was limited, although surgeons experienced substantially more pain complaints after LRP compared to RARP (69% vs 21%) (Supplement C). As similar attempts to incorporate ergonomic differences of interventions on physicians in cost-effectiveness analyses are scarce, we (pragmatically) translated the costs per surgeon having sick leave to costs per patient. In this method the costs for one surgeon having sick leave was divided over ± 38 patients. Although we used the most common approach to incorporate ergonomic effects as financial effect^29^, it could be argued that our approach underestimates its impact, especially when one would adopt a hospital perspective.

The QALY values identified for both interventions were rather high, representing a positive outcome for both treatment options. The QALY difference found, in favor of RARP, was neither statistically nor clinically relevant which is in line with the clinical results where the authors identified no statistically significant difference on overall QALYs measured with the EQ5D-5L^12^. Contrary, they showed a statistically significant and clinically relevant difference on urinary functioning (measured with the EPIC-26^12^). This can be explained by the fact that the EQ5D-5L is not a disease specific questionnaire and therefore less sensitive to specific functional problems. As urinary functioning is an important functional outcome after RP we consider both on the clinical analysis and on the present analysis that the effectiveness is in favor of RARP.

Our findings and conclusions seem to be in line with previous literature showing that RARP was more costly ($7,504–$9,737) compared to LRP ($6,320–$10,991), resulting in ICURs ranging between $28,801–$31,673^21^. Comparison with the findings from another review (including 38 cost-effectiveness studies) was more challenging because in these studies various methods were used to incorporate the costs (e.g. evaluation of the costs based on cost-to-charge ratios or hospital charges) and/or authors only presented incremental costs or savings^11^. However, in general, their results seem to point in the same direction: RARP could be cost-saving when optimal outcomes can be achieved, and the medical equipment is optimally used^11^. Yet, we should note that when the cost-effectiveness of RARP was compared to ORP, RARP is expected to show a smaller chance to be cost-effective, as the costs of ORP are lower compared to LRP^11,21^ but outcomes are expected to be similar to LRP^30^.

The strength of the present analysis is that it is the first analysis comparing RARP to LRP using long-term functional outcome data and incorporating additional care for complaints of incontinence and erectile dysfunction. Besides, this is one of the few analyses adopting a societal perspective^11^, and as far as we know, the first analysis incorporating costs related to homecare and ergonomic complaints of surgeons. A final strength is the bottom-up cost analysis of the intervention and follow-up costs as this provides an accurate and transparent overview of the costs^31^.

Several limitations should be acknowledged. First, the generalizability of our results may be limited by the focus on the Dutch healthcare system. We, therefore, presented all cost input parameters transparently to enable calculation of reliable estimates for other countries as well. Furthermore, the cost-effectiveness of RARP may be underestimated because we had no data on the recovery of functional outcomes in the years after surgery, and the recovery duration was suggested to be in favor of RARP^32,33^. Also we did not include costs of hormonal therapy, although a higher proportion of patients received hormonal treatment after LRP compared to RARP^12^. Contrary, the functional outcomes found for LRP could be underestimated due to the chosen time frame, since the larger hospitals – having more advanced urologists on average – are expected to have shifted earlier to RARP. However, incorporating several confounders in the clinical analysis, did not alter our conclusion^12^, for which we are confident that our results point in the right direction.

We conclude that RARP is cost-effective compared to LRP when evaluating long-term health and economic effects at most acceptable WTP ratios. When RARP is centralized and surgeons are experienced with the Da Vinci robot and/or the Da Vinci robot is used in multiple indications, RARP becomes cost-effective at all WTP ratios and has the potential to be cost-saving. Therefore, our results are a clear incentive to fully reimburse RARP, especially when hospitals provide RARP centralized.

## Supplementary Information

Supplementary Information.

## Data Availability

The datasets used and/or analysed during the current study are available from the corresponding author on reasonable request.

## Abbreviations

ABC: Activity based costing
CEA: Cost-effectiveness analysis
CE: Cost-effectiveness
CEAC: Cost-effectiveness acceptability curve
DSA: Deterministic sensitivity analysis
EPIC-26: Expended prostate index composite short form 26
ICUR: Incremental cost utility ratio
LND: Lymph node dissection
LRP: Laparoscopic radical prostatectomy
ORP: Open radical prostatectomy
ProbSA: Probabilistic sensitivity analysis
RARP: Robot-assisted radical prostatectomy
SHIM: Sexual health inventory for men
WTP: Willingness to pay
