# Supplementary material for: Cost-utility analysis on robot-assisted and laparoscopic prostatectomy based on long-term functional outcomes
Source: Sci Rep. 2022 May 10;12:7658. doi: 10.1038/s41598-022-10746-3 (PMC9090736; doi:10.1038/s41598-022-10746-3)
Supplement: Supplementary file 1 — Supplementary Information. [file 41598_2022_10746_MOESM1_ESM.docx]

# Supplement A – Schematic overview of the model





Supplement A – Decision tree model. Both LRP and RARP could lead patients the similar health states.

# Supplement B – Observed SHIM scores

SHIM scores

|  | RARP (n=840)  Missing: 67 (7.4%) | LRP (n=407)  Missing n=56 (12.1%) |
| --- | --- | --- |
| Severe erectile dysfunction (1-7) | 578 (68.8%) | 317 (77.9%) |
| Moderate erectile dysfunction (8-11) | 92 (11.0%) | 46 (11.3%) |
| Mild to moderate erectile dysfunction (12-16) | 38 (4.5%) | 19 (4.7%) |
| Mild erectile dysfunction (17-21) | 73 (8.7%) | 12 (2.9%) |
| No erectile dysfunction (22-25) | 59 (7.0%) | 13 (3.2%) |

# Supplement C – Surgeon questionnaire

*Participating hospitals CERA-PRO*

*Operating clinicians between 2010-2012*

| You work in: *(name of hospital)* |  | |
| --- | --- | --- |
| Gender: | Man / woman | |
| Age: |  | Years |

***Part 1: Experience***

| 1. When was your first robot-assisted prostatectomy (approximately)? |  | |
| --- | --- | --- |
|  |  | |
| 1. How much experience do you have with the laparoscopic removal of the prostate? |  | Years / Months* |
|  |  |  |
|  |  | Average amount of surgery that you perform(ed) per year / month * |
|  | ** delete what does not apply* | |
| 1. How many robot-assisted procedures do you perform currently on average per year? |  | Per year |
|  |  |  |
| 1. How many robot-assisted procedures did you perform before 2010? |  | Number of procedures  independently before 2010 |
|  |  | Number of procedures while being educated / as a fellow before 2010 |

***Part 2: Ergonomics***

| 1. What proportion of your total work consists of surgical removal of the prostate? |  | % |
| --- | --- | --- |
|  |  |  |

| 1. Do you experience chronical neck or back pain? | - No - Yes, pain in the neck - Yes, pain in the back - Yes pain in both: neck and back |
| --- | --- |

| 1. If yes, do you think that your surgical activities caused the pain or worsen the pain? | - No - Yes these activities caused the pain - Yes, these activities worsened the pain |
| --- | --- |

| 1. Do you or did you experience back or neck pain after a laparoscopic prostatectomy? | - No - Yes, neck pain - Yes, back pain - Yes, neck and back pain - No, but other pain namely: |
| --- | --- |
| 1. Do you or did you experience back or neck pain after a robot-assisted prostatectomy? | - No - Yes, neck pain - Yes, back pain - Yes, neck and back pain - No, but other pain namely: |

| 1. Is your preference for the type of OR (laparoscopic or robot-assisted) influenced by physical complaints? For example by neck and or back pain? | - No this doesn’t matter - Yes a preference because of physical complaints for laparoscopic removal - Yes a preference because of physical complaints for robot-assisted removal |
| --- | --- |

| 1. If you indicated that you experience pain after performing a laparoscopic prostatectomy, which might have caused chronical complaints or worsened existing complaints: | | | |
| --- | --- | --- | --- |
| 1. Did you use care for this? | | - No - Yes | |
| If yes, could you specify below what type of care, the frequency and the duration of this type of care? (medication, physiotherapy, occupational therapy etc.) | | | |
|  | Frequency:  Duration: | |  |
|  |  |  |  |

|  | 1. Did this result in higher sick leave? | - No - Yes |  |
| --- | --- | --- | --- |
|  | If yes, could you specify (in days or weeks) the extend that the sick leave was worsened in days or weeks? | |  |
|  |  | |  |
|  |  | |  |
|  | | | |
| 1. If you indicated that you experience pain after performing a robot-assisted prostatectomy, which might have caused chronical complaints or worsened existing complaints: | | | |
| 1. Did you use care for this? | | - No - Yes | |
| If yes, could you specify below what type of care, the frequency and the duration of this type of care? (medication, physiotherapy, occupational therapy etc.) | | | |
|  | Frequency:  Duration: | |  |
|  |  |  |  |
|  | 1. Did this result in higher sick leave? | - No - Yes |  |
|  | If yes, could you specify (in days or weeks) the extend that the sick leave was worsened in days or weeks? | |  |
|  |  | |  |
|  |  | |  |
|  | | | |

1. According to you what are other advantages for you or for the patient in using the robot in a prostatectomy?

| - For the surgeon: |  |
| --- | --- |
|  |  |
| - For the patient: |  |
|  |  |
| - I don’t see specific advantages but I see the following disadvantages for me / patient / organization: |  |

You completed the questionnaire. Thank you for your participation!

If you have any other remarks regarding the use of the robot for surgical removal of the prostate you can use the space below.

|  |
| --- |

# Supplement D – Results of the surgeon questionnaire

Six of the fourteen surgeons that completed the questionnaire agreed that their surgical activities worsened (n=3) or caused (n=3) chronical neck and/or back pain. After LRP, 9 of 13 (69.2%) responding surgeons described to experience back and/or neck pain, 3 surgeons (23.1%) used additional care for their complaints and for one surgeon it resulted in sick leave. After RARP, 3 of 14 surgeons experienced back or neck pain (21.4%), one surgeon (7.1%) used additional care and for no surgeon it resulted in sick leave. In the table below the characteristics or questions and answers are listed.

| Question or characteristic | | Numbers of surgeons (%) |
| --- | --- | --- |
| **Sex**  Male  Female | | 13  1 |
| **Age** median (y) | | 50.5 (40-62) |
| **Year first RALP performed**  2005.00  2007.00  2008.00  2009.00  2010.00  2011.00  2018.00  Missing | | 1  1  1  1  3  5  1  1 |
| **Years of experience with LRP median** (range) | | 7 (0.5-20) |
| **Number of LRP procedures per year median** (range) | | 40 (5-50) |
| **Number of RALP procedures per year median** (range) | | 80 (0-250) |
| **Current percentage of performing surgeries of total work activities** (median, range) | | 17.5% (0% - 40%) |
| **Do you experience chronical neck or back pain?**  No  Yes, pain in the neck  Yes, pain in the back  Yes pain in both: neck and back | | 7 (23.1%)  3 (23.1%)  2 (15.4%)  1 (7.7%) |
| **If yes, do you think that your surgical activities caused the pain or worsen the pain?**  Yes these activities caused the pain  Yes, these activities worsened the pain | | 3 (50%)  3 (50%) |
| **Is your preference for the type of OR (laparoscopic or robot-assisted) influenced by physical complaints? For example by neck and or back pain?** | | |
| No this doesn’t matter  Yes a preference because of physical complaints for laparoscopic removal  Yes a preference because of physical complaints for robot-assisted removal | | 5 (38.5%)  1 (7.7%)  7 (53.8%) |
|  | LRP | RARP |
| **Do you or did you experience back or neck pain?** | | |
| No  Yes, pain in the neck  Yes, pain in the back  Yes pain in both: neck and back  No, but other pain namely: | 4 (30.8%)  1 (7.7%)  2 (15.4%)  6 (46.2%)  0 | 11 (78.6%)  2 (14.3%)  0  1 (7.1%)  0 |
| **If you indicated that you experience pain after performing a prostatectomy, did you use care for this?** | | |
| Yes  No | 3 (37.5%)  5 (62.5%) | 1 (33.3%)  2 (66.7%) |
| **If you indicated that you experience pain after performing a prostatectomy did this result in higher sick leave?** | | |
| Yes  No | 1 (12.5%)  7 (87.5%) | 0  3 (100%) |

# Supplement E – More detailed description of the calculated costs

**Surgeon effects**

To calculate the costs related to complaints or sick leave of surgeons in the model, the percentage of urologists having sick leave (5%) and using care (20%) stratified for after RARP and LRP were linked to costs of additional care and productivity losses by using the friction cost method[14]. These costs were multiplied with the number of main operating urologists in the 12 hospitals between 2010-2012 (n=36) and the percentage of patients receiving LRP (34.8%) and RARP (65.2%) to incorporate the costs for both interventions.

**The intervention costs**

To calculate the personnel costs, the required personnel was linked to annual loaded salaries based on the collective labor agreements and the Dutch costing manual[14,20,39]. For the operating surgeon, the costs were multiplied with the average skin-to-skin duration of the procedure. The costs for other staff was multiplied to the average procedure time. For the material costs all materials used, including the specific instruments used for RARP, were identified and linked to internal costs. When the cost-analysis was performed, only one of the two LRP hospitals still performed LRP, therefore the material costs were based on literature[7]. All material costs were corrected for inflation[21]. For the medical device costs, the yearly costs of interest and amortization were calculated by using the purchasing costs of the Da Vinci robot, a depreciation period of 10 years, and an interest rate of 4.2%[14]. These yearly costs were divided by the number of prostatectomies per year in each hospital. Overhead expenses, 38% recommended by the Dutch guideline[14], were only calculated over the material costs to avoid double counting. The costs for using the OR were based on a previous study from a Dutch perspective[19] showing that one hour of OR use costs €238.20 without personnel. These costs were multiplied with the average total procedure time.

**Complication costs**

Costs for Grade 1 and 2 consisted of 1 and 2 additional nursing days respectively. For Grade 3, 3 additional nursing days and a general surgery to treat e.g. urine leakage were charged[40]. For a Grade 4 complication, we charged 5 ICU days including diagnostic activities[14]. These numbers were multiplied with the probability of having a Grade 1 to 4 complication after RARP and LRP.

**Home care costs**

For homecare costs, a weighted average of the unit costs for personal care, and nursing care was calculated[14]. These costs were multiplied to the number of patients not receiving homecare before surgery, the average duration, and average frequency[11].

**Health state costs**

To calculate the costs related to being in a certain health state, the evaluated activities were linked to unit costs and the time horizon. For patients being incontinent and impotent, the number of pads used per day at follow-up was evaluated and multiplied with the unit costs and the time horizon. Pad use was assumed to be stable over time. For patients having erectile dysfunction complaints, the percentage of patients initially using specific pharmaceuticals was multiplied with their success rates based on data from the survey and literature, and with the unit cost[12,13,17]. Based on guidelines, it was assumed that a PDE-5 inhibitor was used 3 times a week and Intra-urethral and Intra-cavernous injections twice a week when successful (Table 1).

# Supplement F – Detailed information the input parameters for the scenario analysis

In the table below the parameters that were adjusted compared to the original table 2 are bold. In addition, the transition probabilities for RARP were adjusted by evaluating the 458 number of patients that were operated in the two hospitals having a throughput above 150 cases per year. This resulted in: 18.9% in the continent and potent health state, 57.7% incontinent and impotent health state and 21.4% in incontinent and impotent health state. Compared to 13.03%, 55.64% and 31.33% respectively in the base case.

| **Intervention costs input (for centralization scenario)** | | |  | | |  |
| --- | --- | --- | --- | --- | --- | --- |
|  | **LRP (95% CI)** | **RARP (95% CI)** | | | **Source** | |
| Input for RP without LND |  |  | | |  | |
| Total procedure time (mean hours) | 3.61 (3.53-3.69) | **2.65 (2.57-2.72)** | | | [11] | |
| Skin-to-skin procedure time (mean hours) | 3.06 (2.99-3.12) | **2.04 (1.97-2.11)** | | | [11] | |
| Length of stay (mean days) | 2.99 (2.86-3.13) | **2.69 (2.55-2.84)** | | | [11] RARP based on 252 patients | |
| Input for RP with LND |  |  | | |  | |
| Total procedure time (mean hours) | 4.25 (4.07–4.42) | **3.05 (2.96-3.14)** | | | [11] | |
| Skin-to-skin procedure time (mean hours) | 3.74 (3.60-3.88) | **2.44 (2.36-2.52)** | | | [11] | |
| Length of stay (mean days) | 4.59 (4.03-5.14) | **2.57 (2.41-2.72)** | | | [11] RARP based on 212 patients | |
| Input regardless of with or without LND |  |  | | |  | |
| Percentage of patients receiving LND | 26.8% (23%-31%) | 37.9% (35%-41%) | | | [11] | |
| Costs of OR usage per hour | € 238.20 | € 238.20 | | | [19] | |
| Personnel costs per hour: Anaesthetist (0.5), Surgeon (1-2), OR assistant (2.2), Medical assistant (1) on average per hour | € 366.60 | € 323.66 | | | Real time observation per hospital; Collective labour agreement | |
| Hospitalization costs per day | € 505.32 | € 505.32 | | | [14] | |
|  |  |  | | |  | |
| **Intervention costs results** |  |  | |  |  | |
|  | **LRP** | **RARP** | | | **Source / calculation** | |
| Intervention cost without LND |  |  | | |  | |
| Personnel per procedure | € 1,225.25 | **€ 782.19** | | | Collective labour agreement / internal registry data RARP | |
| OR usage per procedure | € 859.88 | **€ 630.24** | | | [19] | |
| Hospitalization per procedure | € 1,512.97 | **€ 1,361.56** | | | [14] | |
| Material costs (e.g. surgical tools, suture material, Da Vinci materials) | € 2,417.67 | € 2,786.85 | | | LRP: [7]*; RARP: based on internal costs per hospital | |
| Medical devices costs (equipment costs and service costs) | - | **€ 1,805.08** | | | Interviews / internal cost information of 3 hospitals | |
| Overhead | € 918.71 | € 1,059.00 | | | [7,14] | |
| Intervention cost with LND |  |  | | |  | |
| Personnel per procedure | € 1,459.54 | **€ 911.49** | | | Collective labour agreements / internal registry data | |
| OR usage per procedure | € 1,011.32 | **€ 725.95** | | | [19] | |
| Hospitalization per procedure | € 2,317.08 | **€ 1,296.67** | | | [14] | |
| Material costs (e.g. surgical tools, suture material, Da Vinci materials) | € 2,417.67 | € 2,786.85 | | | For LRP: [7]*; RARP based on internal costs | |
| Medical device costs (equipment costs and service costs) | - | **€ 1,805,08** | | | Internal cost information | |
| Overhead costs | € 918.71 | € 1,059.00 | | | [7,14] | |
| Total costs without LND  Total costs with LND  Total costs per intervention (used in the CUA) | € 6,934.48  € 8,124.32  € 7,253.36 | **€ 8,424.92**  **€ 8,585.05**  **€ 8,485.61** | | |  | |
| Table 2 adapted for the scenario analysis – Intervention costs in detail, as used in the scenario analysis. The numbers in bold show the adapted values.  * exchange rate from pound to euro of 1.23 EUR (average rate of 2012) costs were corrected for inflation (1.105 from 2012 to 2019) | | | | | | |
